# Ethnomedicinal uses of plants for the treatment of nervous disorders at the herbal markets of Bojnord, North Khorasan Province, Iran

**Published:** 2019

**Authors:** Mohabat Nadaf, Mohammad Joharchi, Mohammad Sadegh Amiri

**Affiliations:** 1 *Department of Biology, Payame Noor University* *(PNU), Tehran, Iran*; 2 *Department of Botany, Research Center for Plant Sciences, Ferdowsi University of Mashhad, Mashhad, Iran*

**Keywords:** Medicinal plants, Lamiaceae, Frequency of citation, Family importance value

## Abstract

**Objective:**

Since ancient times, plants have been commonly used to cure human ailments by indigenous people throughout the world. Nervous diseases are rising all around the world. This paper provides important ethnobotanical information on plants that are used against neurological disorders and are available in medicinal markets of Bojnord, northeastern Iran.

**Materials and Methods:**

This survey, as an ethnobotanical study, was carried out between 2017 and 2018. The identification of the intended plant species was done using the available Floras. Some important indices such as the frequency of citations, relative frequency of citation (RFC), family importance value and use report were calculated for the medicinal plants included in the present study.

**Results:**

The present study includes 58 medicinal plant species belonging to 36 families and reports their mentioning scientific and vernacular names, parts used, and preparation method. The most common family was Lamiaceae. The major parts of the identified plants used for treatment of nervous )neuropsychological( disorders were flower and aerial parts. The most common methods used for preparation of these plants were infusion and decoction. *Stachys* *turcomanica*, *Tripleurospermum* *disciforme*, *Melissa officinalis*, *Nardostachys jatamansi*, and *Aloysia* *citriodora* had the highest rate of use report. *Echium* *amoenum* and *Melissa officinalis* had the highest RFC.

**Conclusion:**

The study indicated that although people in Bojnord have access to modern medicinal preparations, a considerable number of them still use medicinal plants for therapeutic purposes. Seemingly, most cited plants are worth more precise evaluations for their pharmacological activity.

## Introduction

Indigenous knowledge of medicinal herbs is regarded as an important source of information for health care, throughout the world (Hamilton, 2004 ▶; Reyer-Garcia et al., 2013 ▶). Nowadays, traditional medicinal plants are extensively utilized for treating ailments (Davidson-Hunt, 2000 ▶). Herbal medicine refers to the medical use of plant parts (leaves, stems, root. flowers, fruits and seeds) for treatment of diseases (Iriti et al., 2010 ▶). Most of the population use traditional medicine for health care. As positive aspects of traditional medicine, researchers have mentioned diversity, flexibility, accessibility, relevance in developing countries, relatively low cost, and few side effects of medicinal plants (Payyappallimana, 2010 ▶). Traditional medical knowledge of plant species and their uses by indigenous people are useful not only for conservation of inherited traditional medicine, but also for drug development (Kantati et al., 2016 ▶). It is estimated that nearly 25% of modern drugs are directly or indirectly originated from plants (Imanshahedi and Hosseenzadeh, 2006 ▶). Medicinal plants species have shown therapeutic potentials for treatment of neurological disorders. According to the World Health Organization (WHO) reports, more than one billion people worldwide suffer from illnesses of the central nervous system (CNS). Mental disorders primarily appear as abnormalities of thought, feeling or behavior, producing either distress or impairment of function (calvo and Cavero, 2015 ▶). Depression and anxiety are clinic illnesses related to the CNS. Depression as the second reason of disability after cardiovascular diseases, causes severe social and economic deficits (Saki et al., 2014 ▶).

Depressed people experience periods of reduced productivity. Therefore, many arguments have been put forward in this context (Jelodar et al., 2018 ▶). Anxiety disorders are the most common types of mental illnesses observed in communities. Insomnia is one of the most common disorders that many people are chronically suffering from for different reasons (Neubaur, 2003 ▶). Alzheimer's disease is the most common cause of severe mental deterioration (dementia) in the elderly (Akhonzadeh and Maleki, 2006 ▶; Poorgholam et al., 2018 ▶). Epilepsy is a chronic disorder, estimated to affect at least 10 million people worldwide, and it is associated with alterations in mental processes, state of consciousness or involuntary movements. Convulsion is abnormal discharge of a group of neurons in the CNS, and may occur in different clinical forms depending on the discharge rate and its spread (Saki et al., 2014 ▶). Taking into account the existing knowledge on the medicinal plants used for treatment of neurological disorders, it is necessary to collect and standardize the information available about plant species used for curing the nervous system. Recently, ethnic people or rural communities around the world have gathered such information (Calvo and Cavero, 2015 ▶; Saki et al., 2014 ▶; Kantati et al., 2016 ▶; Sahoo, 2018 ▶; Akhonzadeh and Maleki, 2006 ▶; Maleki and Akhani, 2018 ▶; Mosaddegh et al., 2012 ▶; Dolatkhah et al., 2014 ▶). Nevertheless, most of these studies were conducted qualitatively and lack quantitative analysis of ethnomedicinal data. This paper aims at introducing the ethnoneurological uses of medicinal plants and highlighting candidate plants for further pharmacological investigations. Moreover, we applied some quantitative approaches to analyze folk medicine knowledge available in this field.

## Materials and Methods

**Study area**

Bojnord is located in the northeast of Iran. It is the capital city of North Khorasan province, Iran. It is about 701km from Tehran. Its approximate geographic location is between 57° 17' to 57° 28' eastern longitude and 37° 13' to 37° 50' northern latitude. The total surface area of Bojnord is 36 km^2 ^with a population of about 228931 people. Bojnord has a semiarid and cold climate with annual precipitation ranging between 414.6 to 126.1mm and maximum and minimum temperatures of 19.9 and 6.8°C.

The city is famous for its multicultural background. Many people speak at least 2 of the following languages: Persian, Tati, Khorasani Turkic, Kurmanci Kurdish, and Turkmen. Intermarriage between the ethnic groups speaking these languages is common.

**Data collection and nomenclature**

In order to gather information on medicinal plants used for curing nervous (neuropsychological) disorders that are found in the herbal markets of Bojnord, data were collected during 2017-2018. Thirty-four informants (aging 28-70 years; 30 males and 4 females) in the herbal markets, were interviewed (Table 1).

During the interviews, ethnobotanical information, including vernacular names, utilized parts, purpose of usage, duration of the treatment and the method of preparation were recorded for plants used for curing neuropsychologicalproblems. After data collection, we presented specimens of plants to different people to confirm the accuracy of results. These medicinal plants collected from herbal markets in Bojnord were identified and authenticated by using floristic and taxonomic references, especially Flora Iranica (Rechinger, 1963-2015 ▶), Flora of Iran (Assadi et al, 1988-2013 ▶). Identified species were deposited at the herbarium of Payame Noor university of Bojnord. In this paper, the accuracy of scientific and author names of medicinal plants were confirmed using www.theplantlist.org.

**Data analysis**

One important aspect of quantitative ethnobotanical analysis is the use of quantitative techniques to assess the medicinal use of plants in a specific area (Hussain et al., 2018 ▶). The following indices were calculated in the present study.

**Frequency of Citation (FC) and relative frequency of citation (RFC)**

The frequency of citations (FC) is the number of informants who mentioned a certain species.

Relative frequency of citation (RFC) is obtained by dividing frequency citation (FC) by total number of informants in the survey (N). The value of RFC for species of medicinal plants is based on the citing percentage of informants for every species. RFC was calculated by using the following formula (Tardio and Pardo-de Santayana, 2008 ▶):

RFC=FC/N (0<RFC<1)

RFC varies from zero (when nobody refers to a plant as a useful one), to one (when all the informants consider a certain species beneficial).

**Use report (UR)**

Use report (UR) is the use recorded for every plant species in this survey.

**Family importance value (FIV)**

Family importance value (FIV) gives the local importance of the families of wild species. It was calculated by calculating the percentage of informants mentioning a specific family (Vitalini et al., 2013 ▶). 

FIV=FC (family)/ N×100

Where FC is the number of informants mentioning the family and N is the total number of informants participated in the study.

**Statistical analyses**

All statistical analyses are carried out with Microsoft Excel 2016.

## Results

**Diversity of medicinal plants**

A total of 58 medicinal plants used for treatment of neuropsychological disorders were identified at the herbal markets in Bojnord. Information of identified plants obtained from this survey is shown in Table 2. Ethnobotanical data, scientific name, family, local name, parts used, preparation methods, frequency of citation (FC), number of use reports (UR) and therapeutic applications are represented for each plant species.

**Figure 1:**
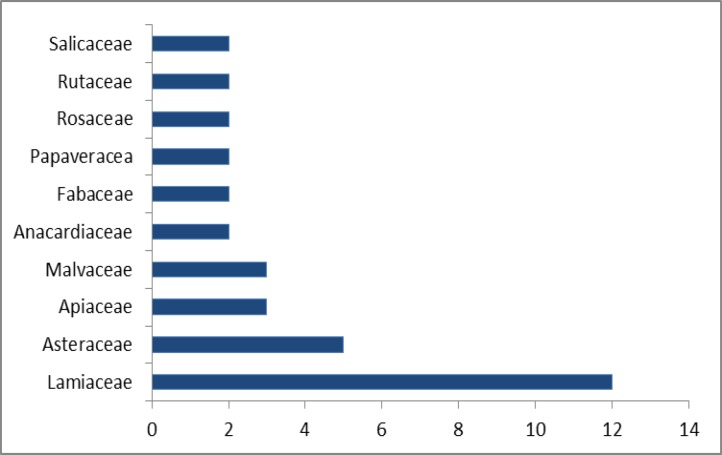
Plant families with the highest number of cited species for the treatment of neuropsychological disorders

The 58 medicinal species belong to 54 genus and 36 families namely, Lamiaceae (10 species), Asteraceae (5 species), Apiaceae (3 species), Anacardiaceae (2 species), Fabaceae (2 species), Rosaceae (2 species), Rutaceae (2 species), Salicaceae (2 species) and Verbenaceae (2 species). The other families were represented by only one species (Table 2 and Figure 1).

**The plant parts used and the method of preparation**

**Figure 2:**
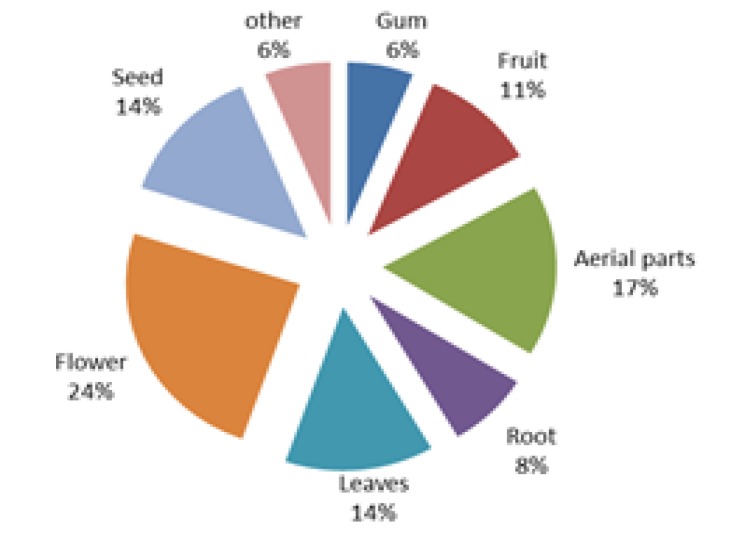
The frequency of application of different parts of medicinal plants used for treatment of neuropsychological disorders

Different parts of medicinal plants were used to cure neurological disorders. Among them, the most frequently used parts were flower (24%) and aerial parts (17%), followed by leaves (14%), seeds (14%), fruits (11%), root (8%), gum (6%) and other parts (6%) (Figure 2).

**Table 1:** Demographic data of the interviewed informants at the herbal markets of Bojnord, North Khorasan Province, Iran

**Variable**	**Demographic** **catrgories**	**Number of** **informants**	**Percentages**
**Gender**	Female Male	4 30	11.76 88.23
**Experience**	Local healers	34	100
**Age groups**	20-40 41-60 Above 60	9 18 7	26.4 61.76 20.5
**Education**	Diploma Bachelor Masters	7 24 3	20.5 70.5 8.8
**Residence**	Urban	34	100

**Figure 3:**
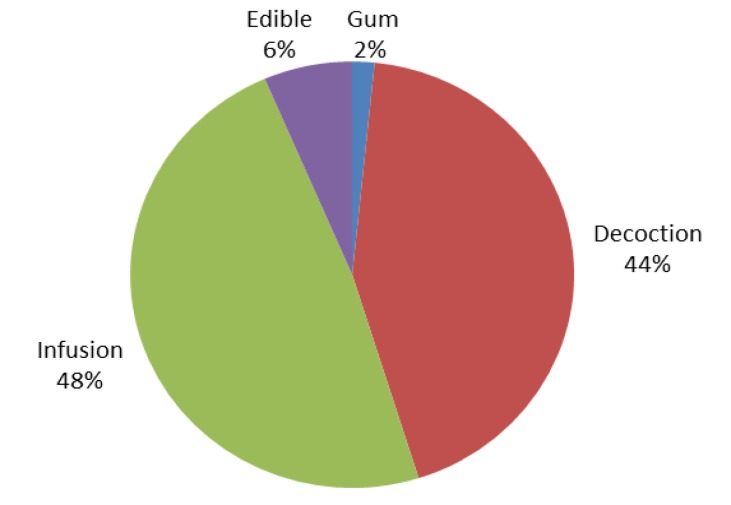
Frequency of different preparations of medicinal plants used for treatment of neuropsychological disorders. Quantitative analysis of data

 The preparations are divided into 4 categories including chewing gum, decoction, infusion, and edible. The most important preparations were infusion (48%) and decoction (44%), followed by edible (6%), and chewing gum (2%) (Figure 3).

**Family importance value (FIV)**

**Figure 4:**
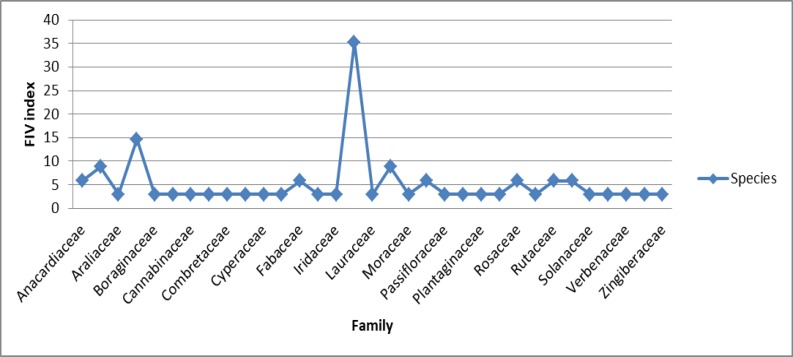
Family importance value (FIV) of medicinal plants used for treatment of neuropsychological disorders

The most common family of medicinal plant species, based on the number of species and FIV index, was Lamiaceae (12 species with FVI 35.29) followed by Asteraceae (5 species with FVI 14.70 FIV), Apiaceae and Malvaceae (3 species with FVI 8.82 FIV), and Anacardiaceae, Fabaceae, Papaveraceae, Rosaceae, Rutaceae and Salicaceae (2 species with FVI 5.88 for each). The other families had lower number of species and (1 species with FVI 2.94), (Figures 1 and 4).

**Use report (UR), frequency of citation (FC) and relative frequency of citation (RFC)**

*Echium* *amoenum* and *Melissa officinalis* (FC 24 for both) had the maximum citation in this study (Table 2).

**Figure 5:**
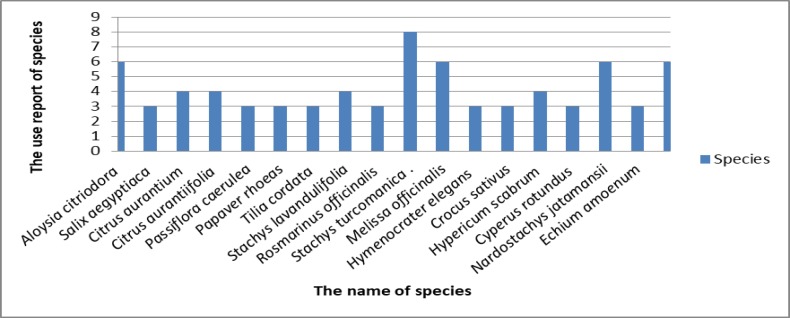
Use report for medicinal plants applied for treatment of neuropsychological disorders

*Stachys* *turcomanica *Trautv (UR 8), *Tripleurospermum* *disciforme *(C.A.Mey.) Sch.Bip. (UR 6), *Melissa officinalis* L. (UR 6), *Nardostachys* *jatamansii *(D. Don) DC. (UR 6), *Aloysia* *citriodora *Palau*. *(UR 6) had the highest use report (UR) (Table 2 and Figure 5).

**Figure 6:**
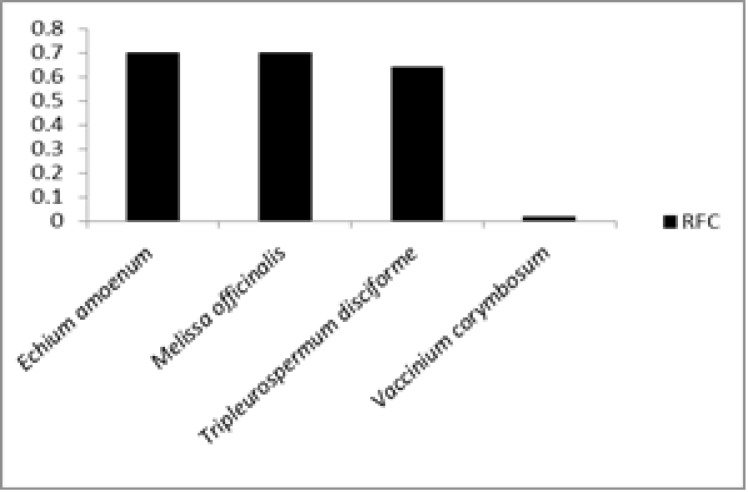
Plant species with highest and lowest RFC

Highest RFC was calculated for *Echium* *amoenum* and *Melissa officinalis* (0.7 for both) followed by *Tripleurospermum* *disciforme* (C.A.Mey.) Sch.Bip. (0.64) and the least RFC was calculated for *Vaccinium arctostaphylos* L. (0.02) (Table 2 and Figure 6). 

**Table 2:** Medicinal plants and their uses for treatment of neuropsychological disorders

**NO**	** Family Name**	***Scientific name***	**Local name**	**Part used**	**Uses**	**Preparation**	**FC**	**RFC**	**UR**
**1**	Anacardiaceae	*Pistacia* *atlantica *Desf. subsp. Kurdica	"Saghez"	Oleo-gum-resin	to strengthen the memory	Chewing gum	6	0.17647	1
**2**		*Pistacia* *lentiscus *L.	"Mastaki"	Gum	to strengthen the memory	Decoction	10	0.29412	1
**3**	Apiaceae	*Coriandrum* *sativum *L.	"Geshniz"	Fruit	Hypnotic	Infusion	18	0.52941	1
**4**		*Eryngium *sp*.*	"Zolangon,Bughnagh"	Aerial parts	Sedative	Decoction	3	0.08824	1
**5**		*Ferula gummosa *Bioss*.*	"Ghasni"	Root- Gum	Sedative, to strengthen the memory	Decoction	13	0.38235	2
**6**	Araliaceae	*Panax ginseng *C.A.Mey.	"Jin Sing"	Root	Nerve tonic	Decoction	9	0.26471	1
**7**	Asteraceae	*Achillea santolinoides *subsp. *wilhelmsii *(K.Koch) Greuter.	"Bumadaran"	Aerial parts	Nerve tonic	Infusion	20	0.58824	1
**8**		*Artemisia dracunculus *L.	"Talkhan,Tarkhun"	Leaves	Nerve tonic	Decoction, Edible	7	0.20588	1
**9**		*Calendula officinalis *L.	"HamisheBahar"	Flower	Sedative	Infusion	10	0.29412	1
**10**		*Stevia rebaudiana *(Bertoni) Bertoni.	"Estevia"	Leaves	Sedative, Nerve tonic	Infusion	2	0.05882	2
**11**		*Tripleurospermum disciforme *(C.A.Mey.) Sch.Bip*.*	"BabunehDashti"	Flower	Nerve tonic, Antimigraine, Antistress, Sedative, Hypnotic	Infusion	22	0.64706	6
**12**	Boraginaceae	*Echium amoenum *Fisch. & C.A.Mey.	"Gavzaban"	Flower	Nerve tonic, Antistress, Hypnotic	Decoction	24	0.70588	3
**13**	Burseraceae	*Boswellia sacra *Fluek.	"Kondor"	Gum	to strengthen the memory	Infusion	20	0.58824	1
**14**	Cannabinaceae	*Humulus lupulus *L.	"Razak"	Hops	Sedative	Decoction	3	0.08824	1
**15**	Caparifoliaceae	*Nardostachys jatamansii *(D.Don) DC.	"Sonbolotib"	Root	Nerve tonic, Sedative, Hypnotic, Antistress, Antimigraine, Anticonvulsion	Infusion	18	0.52941	6
**16**	Combretaceae	*Terminalia chebula *Retz*.*	"HalilehSiah"	Fruit	Antialzheimer	Decoction	13	0.38235	1
**17**	Convolvolaceae	*Cuscuta epithymum* Murray.	"Aftimun"	Aerial parts	Nerve tonic, Anticonvulsion	Infusion	9	0.26471	2
**18**	Cyperaceae	*Cyperus rotundus* L*.*	"SoadeKufi"	Root	Sedative, to strengthen the memory, Antimigraine	Decoction	5	0.14706	3
**19**	Ericaceae	*Vaccinium* *arctostaphylos *L.	"Belobery"	Fruit	to strengthen the memory	Edible	1	0.02941	1
**20**	Fabaceae	*Arachis hypogaea* L.	"Badamzamini"	Seed	Antialzheimer	Infusion	4	0.11765	1
**21**		*Glycyrrhiza glabra *L*.*	"Shirin Bayan"	Root	Antidepressant	Infusion	19	0.55882	1
**22**	Hypericaceae	*Hypericum scabrum *L.	"Saregul"	Flower	Antimigraine, Antidepressant, Sedative	Infusion	7	0.20588	4
**23**	Iridaceae	*Crocus sativus *L*.*	"Zaffaron"	Stigma	Nerve tonic, Antidepressant, Sedative	Decoction, Edible	9	0.26471	3
**24**	Lamiaceae	*Dracocephalum lindbergii *Rech.f.	"Aghbash"	Aerial parts	Sedative, Hypnotic	Infusion	12	0.35294	2
**25**		*Hymenocrater elegans *Bunge.	"Sephtalek"	Aerial parts	Hypnotic, Antistress, Sedative	Infusion	15	0.44118	3
**26**		*Melissa officinalis *L.	"Badranjbuyeh"	Flower ـ Leaves	to strengthen the memory, Nerve tonic, Hypnotic, Antistress, Sedative, Anticonvulsion	Infusion	24	0.70588	6
**27**		*Lavandula angustifolia *Mill.	"Lavand"	Aerial parts	Nerve tonic	Infusion	2	0.05882	1
**28**		*Mentha longifolia *(L.) Hudson	"Puneh"	Aerial parts	Sedative	Infusion	21	0.61765	1
**29**		*Stachys turcomanica *Trautv*.*	"Ostokhodus"	Aerial parts	Antidepressant, Sedative, Antistress, Antimigraine, Sedative, Anticonvulsion, Nerve tonic, Hypnotic	Infusion	21	0.61765	8
**30**		*Ocimum basilicum *L*.*	"Tokhm Sharbati"	Seed	Hypnotic, Nerve tonic	Infusion	4	0.11765	2
**31**		*Origanum vulgare *L*.*	"Marzanjush"	Aerial parts	Sedative, Nerve tonic	Infusion	18	0.52941	2
**32**		*Rosmarinus officinalis *L*.*	"Rozmari"	Leaves ـ Flower	Antidepressant , Nerve tonic , Hypnotic	Infusion	13	0.38235	3
**33**		*Stachys lavandulifolia *Vahl.	"Ghellecahi"	Flower	Antidepressant, Sedative, Hypnotic, Nerve tonic	Infusion	16	0.47059	4
**34**		*Vitex negundo *L.	"FelfelKuhi"	Flower	Antidepressant	Infusion	6	0.17647	1
**35**		*Zataria multiflora *Boiss.	"AvishanShirazi"	Aerial parts	Nerve tonic	Infusion	23	0.67647	1
**36**	Lauraceae	*Cinnamomum zeylanicum *Nees.	"Darchin"	Bark	to strengthen the memory	Decoction	20	0.58824	1
**37**	Malvaceae	*Theobroma cacao *L.	"Kakau"	Seed	Sleep reducer	Decoction	9	0.26471	1
**38**		*Tilia cordata* Mill.	"Bargtyiol, Zirfun"	Fruit- Leaves	Nerve tonic, Hypnotic, Sedative	Decoction	6	0.17647	3
**39**		*Alcea *spp*.*	"Khatmi"	Flower	Sedative	Infusion	20	0.58824	1
**40**	Moraceae	*Ficus carica *L*.*	"Anjir"	Fruit	Nerve tonic	Decoction, Edible	5	0.14706	1
**41**	Papaveraceae	*Fumaria vaillantii *Loisel*.*	"Shatareh"	Aerial parts	Sedative, Nerve tonic	Decoction	13	0.38235	2
**42**		*Papaver rhoeas *L*.*	"Shaghayegh"	Flower	Addiction, Sedative, Hypnotic	Decoction	2	0.05882	3
**43**	Passifloraceae	*Passiflora caerulea *L*.*	"GolSaati"	Flower	Antidepressant, Hypnotic, Sedative	Decoction	5	0.14706	3
**44**	Pedaliaceae	*Sesamum indicum *L*.*	"Konjed"	Seed	to strengthen the memory	Decoction	17	0.5	1
**45**	Plantaginaceae	*Plantago major *L.	"Barhang"	Seed- Leaves	Antialzheimer	Infusion	17	0.5	1
**46**	Poaceae	*Avena sativa *L	"Jo dosar"	Seed	Antistress, Hypnotic	Infusion	6	0.17647	2
**47**	Rosaceae	*Cydonia oblonga *Mill*.*	"BehDaneh"	Seed- Leaves	Sedative	Decoction	7	0.20588	1
**48**		*Rosa *x* damascene *Mill*.*	"GolMohamadi"	Flower	Antialzheimer, Sedative	Decoction	19	0.55882	1
**49**	Rubiaceae	*Coffea Arabica *L*.*	"Ghahveh"	Seed	Sleep reducer	Infusion	14	0.41176	1
**50**	Rutaceae	*Citrus aurantiifolia *(Christm.) Swingle	"LimuAmani"	Fruit	Sedative	Infusion	17	0.5	4
**51**		*Citrus aurantium *L*.*	"BaharNaranj"	Flower	Sedative , Antimigraine, Antistress, Hypnotic	Decoction	20	0.58824	4
**52**	Salicaceae	*Salix alba *L*.*	"Bid"	Bark- Leaves	Sedative	Decoction	4	0.11765	1
**53**		*Salix aegyptiaca *L*.*	"Bidmeshk"	Flower	Antidepressant, Antimigraine, Sedative	Decoction	4	0.11765	3
**54**	Solanaceae	*Hyoscyamus niger *L*.*	"Bangdaneh"	Seed	Sedative, Addiction	Decoction	3	0.08824	2
**55**	Theaceae	*Camellia sinensis *(L.) Kuntze	"Chaisefid"	Leaves	Sedative	Infusion	5	0.14706	1
**56**	Verbenaceae	*Aloysia citriodora *Palau.	"BehLimu"	Leaves	to strengthen the memory, Anticonvulsion, hypnotic, Sedative, Nerve tonic	Decoction	17	0.5	6
**57**	Violaceae	*Viola odorata *L*.*	"Banafsheh"	Flower	Sedative	Infusion	10	0.29412	1
**58**	Zingiberaceae	*Elettaria cardamomum *Maton.	"Hel"	Fruit	Sedative, Nerve tonic	Decoction	19	0.55882	2

**Effects of traditional treatment and its number**

**Figure 7:**
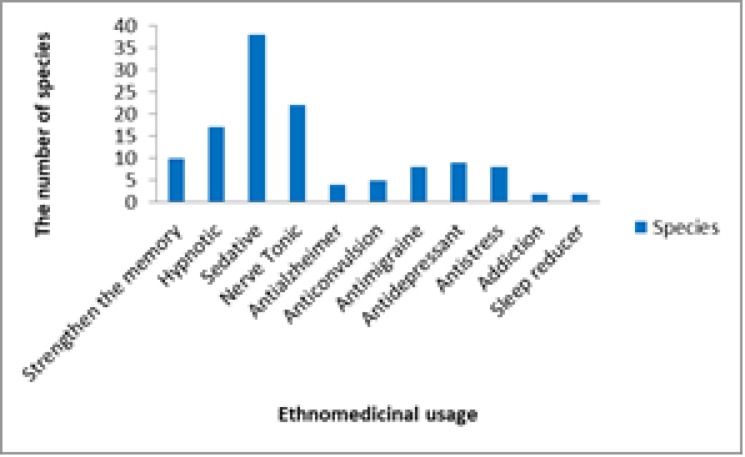
Ethnomedicinal uses of medicinal plants along with the number of plants used for each therapeutic purpose

The highest number of plant species were used as sedative (38 species) followed by Nerve Tonic (22 species). Most of the effects of traditional treatment and its number is shown in Figure 7.

## Discussion

In this study, plant species applied for curing neuropsychological disorders, were summerized.

From the 58 medicinal plants species reported in this paper, some were introduced by other researchers. Among them, *Citrus aurantiifolia* (Christm.) Swingle, *Avena sativa* L., *Panax ginseng *C.A.Mey., *Hyoscyamus niger* L., *Humulus lupulus* L., *Lavandula angustifolia* Mill., *Melissa officinalis* L., *Rosmarinus officinalis* L., *Tilia cordata* Mill., *Ocimum basilicum* L., *Stachys lavandulifolia *Vahl., *Papaver rhoeas *L., *Cydonia oblonga* Mill., *Crocus sativus* L., *Echium amoenum *Fisch. & C.A.Mey, *Mentha longifolia* (L.) Hudson and *Fumaria vaillantii *Loisel., can be named (Farhadi et al., 2016 ▶; Akhonzadeh and Maleki, 2006 ▶; Calvo and Cavero, 2015 ▶; Saki et al., 2014 ▶; Kantati et al., 2016 ▶; Sahoo, 2018 ▶).

Figures 1 and 4 show that the two families of Lamiaceae and Asteraceae have the highest number of cited species and highest rates of FIV. The dominance of these two families is mainly due to some especial properties such as secondary metabolites. Lamiaceae in Iran with 46 genera and 410 species and subspecies, has a great diversity and distribution. Based on the report presented by Naghibi et al. (2005) ▶, 18% of the species of Lamiaceae are used for medicinal purposes. This order of the most used families is similar to a previously published ethnobotany report of Navarra in north of the Iberian Peninsula (Calvo and Cavero, 2015 ▶). Asteraceae was found as the most abundant family in many previous projects like Saki et al. (2014) ▶. It seems that more plants from Asteraceae family have been used for medicinal purposes in comparison with other plant families because of the presence of a wide range of biologically active compounds which produce medicinal effects and also because Asteraceae is one of the largest families in the plant kingdom (Thomas et al., 2009 ▶).

In this study flower and aerial parts of medicinal plants were found to be the most used parts for treatment of neuropsychological disorders (Figure 2). This observation showed that flowering and aerial parts are the preferential sites of accumulation of biologically active compounds. The use of flowering and aerial parts is recommended for sustainability of plant communities (Sadeghi et al., 2014 ▶). Aerial and flowering parts are active in photosynthesis and metabolite production and are also easily accessible and possibly due to these reasons, they were reported as the most widely used part of plant in different areas of Iran (Ghorbani, 2005 ▶). The results of widespread use of infusion and decoction (Figure 3) agree with those reported by Saki et al. (2014) ▶ and Calva and Cavero (2015) ▶ which showed that decoction and infusion were the most commonly used preparation method. Decoction was the most commonly used method for preparation of medicinal plants made by boiling a specific part in water until up to half reduction of water volume. Infusion is the soaking of medicinal plant part in hot water overnight or during day time. Water is the dominant agent used for preparation of herbal medicines (Sadeghi et al., 2014 ▶).

Table 2 and Figure 6 show *Echium amoenum *Fisch. & C.A.Mey. and *Melissa officinalis *L. (FC 24 and RFC 0.7, for both) have the maximum citation in the treatment of neuropsychological disorders. These species had the highest RFC index. Because these plants were mentioned by a large number of informants and RFC directly depends on the number of informants mentioning the use of a specific plant, these species are the most recognized plant in the market ofBojnord. Akhonzadeh and Maleki (2006) ▶ and Farhadi et al. (2016) ▶ reported that these species improve neuropsychological disorders. The highest use reports were found for *Stachys turcomanica *Trautv (8 UR). It was impossible to compare the quantitative data from the market of Bojnord with those of other markets of North Khorasan Province about medicinal plants used for treatment of neuropsychological disorders, as the present study is the first quantitative ethnobotanical report in the market of the region; nationwide, few quantitative works have been done in this regard (Eslami and Khodavari, 2016 ▶; Akhonzadeh and Maleki, 2006 ▶; Saki et al., 2014 ▶).

In this survey, most of plants (38 species) were consumed as sedative agents (Figure 7). Saki et al. (2014) ▶ investigated the medicinal plants used for psychiatric and neurological disorders in Urmia, northwest of Iran. They detected 21 species and showed that most of plants were used as a tranquilizer.

**Figure 8:**
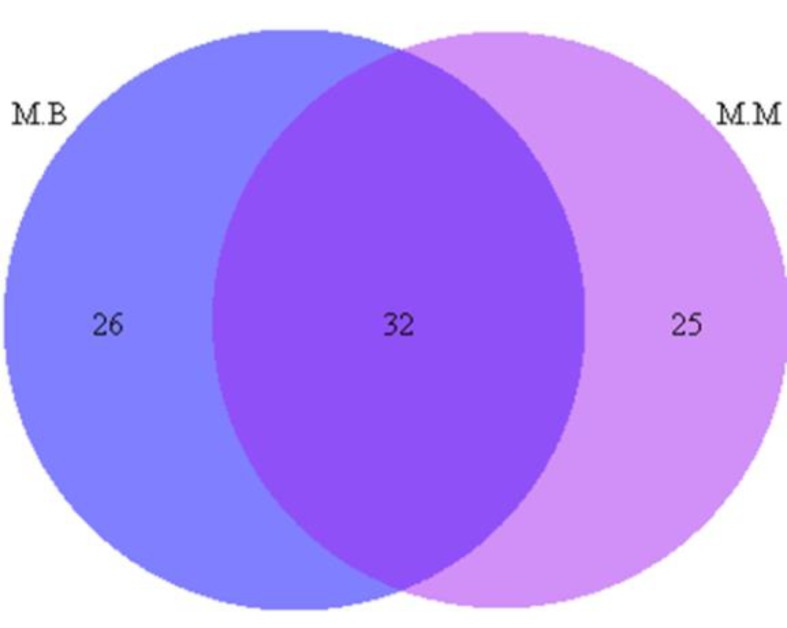
Venn diagram showing the number of medicinal plants used for treatment of neuropsychological disorders in two adjacent cities in northeast of Iran, M.B: the market of Bojnord, M.M: the market of Mashhad

Based on the available information, the presence of 58 medicinal plants used for treatment of neuropsychological disorders, in the markets of Bojnord and 57 medicinal plant species in adjacent markets of Mashhad, the Venn diagram is drawn (Figure 8) which shows all medicinal plants used for treatment of neuropsychological disorders in these two regions. These 2 regions, shared 32 common species.

The present research is the first one done in the markets of Bojnord to assess plants used against neurological and mental disorders. The present study revealed that the area has a variety of medicinal plants and these plants are still commonly used for medicinal purposes by the local inhabitants. However, the new generation is less aware of traditional uses of these plants. Thus, it is important to document and reconstitute the remainders of the ancient medical practices which exist in the area and other parts of Iran, and preserve this knowledge for future generations. The results indicate that traditional healers used 58 species of medicinal plants to cure neuropsychological disorders. Numerous ethnobotanical studies suggest that these medicinal plants have proven to be effective for neurological and mental disorders. It appears that most cited plants are worth more precise evaluation for their pharmacological activities. This study also provided basis for conservation of the medicinal plants.

Iran, with a great experience in using traditional medicinal plants, is an important country in this field and has a growing potential in preparation, production, and export of pharmaceutical plants (Eslami and Khodavari, 2016 ▶)
